# Enhancing Astaxanthin Production in *Paracoccus marcusii* Using an Integrated Strategy: Breeding a Novel Mutant and Fermentation Optimization

**DOI:** 10.3390/md24010019

**Published:** 2026-01-01

**Authors:** Yu Li, Shuyin Huang, Dong Wei, Siyu Pan

**Affiliations:** 1 Guangdong Province Key Laboratory for Green Processing of Natural Products and Product Safety, Engineering Research Centre of Starch and Vegetable Protein Processing Ministry of Education, School of Food Science and Engineering, South China University of Technology, 381 Wushan Road, Guangzhou 510641, China; liyu951020@163.com (Y.L.); sueeyin@163.com (S.H.); 2 Yunnan Asxan Biotech Co., Ltd., 2299 Haiyuan North Road, High-Tech Industry Development Zone, Kunming 650101, China; siyu.pan@asxan.com

**Keywords:** astaxanthin, *Paracoccus marcusii*, compound mutagenesis, microbial microdroplet culture, response surface methodology

## Abstract

Astaxanthin, one of the most commercially valuable carotenoids, is renowned for its potent antioxidant and anti-inflammatory properties and is experiencing growing demand across diverse industries. To enhance astaxanthin production in *Paracoccus marcusii*, compound mutagenesis was performed using ethyl methanesulfonate (EMS), ultraviolet (UV) radiation, and atmospheric room temperature plasma (ARTP) treatment. Subsequently, a high-throughput microbial microdroplet culture (MMC) system was employed to select fast-growing microdroplet, followed by screening for high astaxanthin-producing mutants on dual-inhibitor plates. The mutant M21 was isolated and exhibited a significant increase of 16.86% in astaxanthin content (1.53 mg/g) and a 19.81% increase in astaxanthin production (11.71 mg/L) compared with the wild type (WT) (*p* < 0.05). Moreover, the enhanced phenotype of M21 was genetically stable. Response surface methodology (RSM)-based optimization of fermentation conditions further increased astaxanthin content and production to 1.72 mg/g and 12.92 mg/L, respectively, corresponding to improvements of 16.44% and 23.02% over the WT, while simultaneously reducing culture time, total nitrogen requirements, and sodium lactate consumption, thereby lowering production costs. This study achieved significant enhancement of astaxanthin production through novel mutant breeding and fermentation optimization, underscoring the effectiveness of this integrated strategy for application in industrial biotechnology.

## 1. Introduction

Astaxanthin (C_40_H_52_O_4_) is a lipid-soluble, reddish-orange carotenoid renowned for its exceptional antioxidant capability, which is largely attributed to its terminal ring moiety that effectively traps free radicals [1]. Its antioxidant capacity is approximately 65 times greater than that of vitamin C, 54 times higher than β-carotene, and 100 times greater than α-tocopherol (Vitamin E) [2]. Accordingly, astaxanthin exhibits potent antioxidant, hypoglycemic, immunomodulatory and anticancer activities, and also contributes to muscle and egg yolk pigmentation, enabling its widespread application in the food, cosmetic, nutraceutical, and aquaculture industries as one of the most commercially natural carotenoids [3]. Currently, synthetic astaxanthin dominates the global astaxanthin market, surpassing natural astaxanthin derived from microbial fermentation or extraction from crabs and shrimps [4]. Synthetic astaxanthin is chemically synthesized and exists as a racemic mixture of optical isomers with a ratio of 1:2:1 (3S, 3′S: 3R, 3′S: 3R, 3′R), exhibiting markedly lower antioxidant capacity compared to natural astaxanthin [5]. The free radical inhibition efficiency of natural astaxanthin per milligram is nearly 20 times higher than that of synthetic astaxanthin. The high antioxidant activity and stability of natural astaxanthin are primarily attributed to its degree of esterification with fatty acids, whereas synthetic astaxanthin exists mainly in free, non-esterified form [4].

Several industrial microorganisms, including the microalga *Haematococcus lacustris*, *Ettlia carotinosa*, *Dunaliella salina*, *Chromochloris zofingiensis*, *Chloromonas krienitzii*, as well as the marine yeast *Xanthophyllomyces dendrorhous* and the bacterium *Paracoccus* spp., are promising producers of natural astaxanthin [6,7]. *H. lacustris* can accumulate astaxanthin up to 4.0–7.0% of dry weight (DW); however, its photoautotrophic cultivation is constrained by prolonged growth cycles and stringent culture conditions, resulting in high production costs that limit its commercial application [8]. Astaxanthin derived from *X. dendrorhous* is predominantly the (3R, 3′R)-enantiomer, which exhibits comparatively lower antioxidant activity [9]. To address the limited supply of natural astaxanthin, *Paracoccus* spp., particularly *P. carotinifaciens*, *P. haeundaensis*, and *P. marcusii*, have emerged as a promising microbial source [10]. These bacteria combine short growth cycles with the capacity for high-cell-density fermentation, making them highly suitable for scalable industrial bioprocessing [11]. Notably, *Paracoccus* spp. primarily synthesize the (3S, 3′S)-astaxanthin enantiomer, which possesses superior antioxidant activity and a well-established safety profile. These attributes position *Paracoccus* spp. as a viable cell factory alongside conventional microalgal and yeast platforms [12]. However, the key challenge at present is to increase the astaxanthin content in *Paracoccus* spp.

Traditional mutagenesis techniques, such as chemical mutagenesis (e.g., ethyl methanesulfonate [EMS]) and physical mutagenesis (e.g., UV irradiation), have been widely employed to enhance the production of astaxanthin in *Paracoccus* spp. due to their simplicity and capacity to generate genetically diverse mutant libraries [13,14,15]. For example, the astaxanthin production of *Paracoccus* sp. strain N-81106 was significantly improved to 16 mg/L (17-fold) through random mutagenesis with EMS [16]. Similarly, astaxanthin production in *Paracoccus* sp. LL1 reached 10.2 mg/L, representing a 2.76-fold increase following EMS-induced mutagenesis [12]. Carotenoid biosynthesis inhibitors such as diphenylamine and β-ionone have also been utilized for mutant screening [17]. Nevertheless, the commercialization of *Paracoccus* spp. is still constrained by low astaxanthin productivity and the high costs associated with fermentation [3]. Consequently, integrating strain development with the optimization of fermentation conditions represents an effective strategy to further enhance industrial astaxanthin production. Recently, atmospheric room temperature plasma (ARTP) mutagenesis has emerged as a novel technology based on radio-frequency atmospheric-pressure glow discharge plasma. ARTP induces extensive DNA damage and structural alterations in oligonucleotides, resulting in a significantly higher positive mutation rate compared to traditional methods like UV or chemical mutagenesis at room temperature [18]. Furthermore, the integration of ARTP with the microbial microdroplet culture (MMC) system has proven effective in enhancing biomass and high-value metabolite production across various bacterial, fungal, and algal systems [19].

In this study, an integrated approach to enhance astaxanthin production by *P. marcusii* was developed by generating mutants through EMS-UV-ARTP compound mutagenesis, followed by high-throughput cultivation and selective isolation of mutants. Fermentation conditions were subsequently optimized to further improve astaxanthin production. This combined approach enables strain improvement in *P. marcusii* without the need for genetic engineering, while fermentation optimization provides the necessary nutritional support for the mutant to achieve significantly, elevated astaxanthin production. These strategies present a sustainable and cost-effective solution for utilizing *P. marcusii* mutants in enhanced astaxanthin production.

## 2. Results and Discussion

### 2.1. Optimal Conditions Determination of EMS-UV-ARTP Compound Mutagenesis

**Figure 1:**
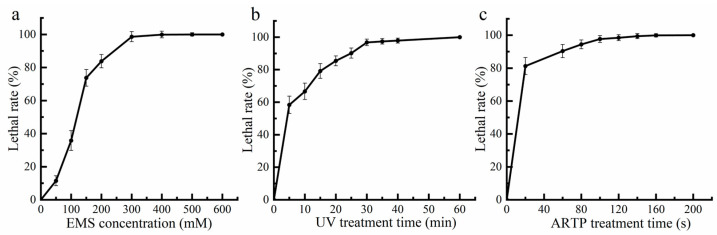
Lethal rate of *P. marcusii* 1.8602 under gradient concentrations of EMS (**a**), time of UV radiation (**b**) and ARTP treatment (**c**).

To determine the optimal conditions for EMS-UV-ARTP compound mutagenesis, cultures in the logarithmic growth phase were treated accordingly and incubated to establish a lethal rate curve. As shown in Figure 1, the lethal rate increased linearly with the dosages (reagent concentration of EMS, or treatment time of UV and ARTP) when the lethal rate was below 90% for all three methods. The dosages required to achieve a 100% lethal rate were more than double those needed for a 90% lethal rate. Previous studies have reported a positive correlation between lethality and mutation rate, indicating that higher lethality enhances the mutational capacity of the strain. A lethal rate exceeding 90% has been shown to yield the highest mutation frequency and consequently the most effective mutagenic outcome [20,21]. Therefore, to maximize the mutation rate while maintain sufficient viability of strains, conditions corresponding to an approximate 90% lethal rate were employed in subsequent mutagenesis. The optimal parameters were determined to be 250 mM for EMS concentration, 25 min for UV exposure time, and 60 s for ARTP treatment.

### 2.2. High-Throughput Culture in the MMC System for Top Microdroplets Screening

**Figure 2:**
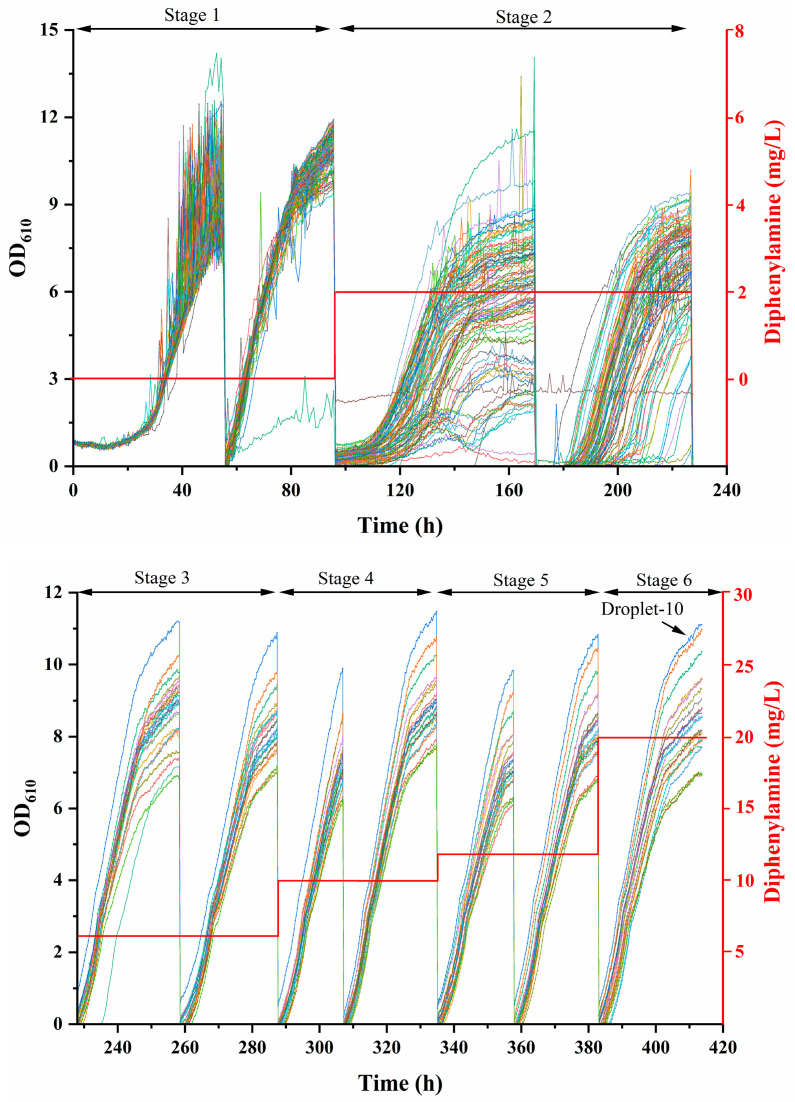
Adaptive culture of microdroplets following compound mutagenesis under gradient diphenylamine concentrations in the MMC system. The red line indicates the diphenylamine concentration.

To efficiently screen mutants exhibiting rapid growth and enhanced astaxanthin biosynthesis, selective culture was conducted automatically in a microdroplet format within the MMC system. As shown in Figure 2, a total of 100 microdroplets underwent six stages of culture, with two successive subcultures performed at each stage and a gradual increase in diphenylamine concentration (0–20 mg/L). In stage 1 (0–96 h), no diphenylamine was added. During the first 24 h after inoculation into the MMC system, cells remained in the lag phase to adapt to the new environment, subsequently entering the logarithmic phase and reaching an OD_610_ of 12 after 60 h. Following one subculture, no evident lag phase was observed, and cell growth increased sharply. During this period, OD_610_ reached 12 in only 36 h, indicating successful adaptation to the MMC environment and readiness for the next stage. The large area-to-volume ratio of microdoplets in the MMC system enhances mass transfer and oxygen availability, thereby supporting rapid microbial growth [22]. In stage 2 (96–228 h), the diphenylamine concentration was increased to 2.0 mg/L through auto-pumping and mixing in the MMC system. The lag phase was prolonged, and cell growth markedly decreased, with considerable variations observed among different microdroplets. Diphenylamine is an inhibitor of carotenoid biosynthesis that primarily targets phytoene desaturase, the enzyme responsible for catalyzing the conversion of phytoene to lycopene, an early rate-limiting step in astaxanthin synthesis. Consequently, exposure to diphenylamine impedes pigment formation and inhibits cell growth [23]. Following an additional, the heterogeneity among microdroplets became more pronounced. Microdroplets exhibiting poor growth performance were discarded, while the remaining microdroplets underwent further subculturing. In stage 3 (228–282 h), no apparent lag phase was observed, and cell growth increased under 2.5 mg/L diphenylamine, indicating that the microdroplets had developed enhanced tolerance. The diphenylamine concentration was gradually increased in the subsequent three stages, with the same culture and subculturing procedures repeated. Notably, microdroplets were ranked according to their OD_610_ value, and only the top microdroplet exhibiting the highest OD_610_ value was collected for further verification. Ultimately, microdroplet-10 demonstrated the highest growth rate (0.87 h^−1^) and maximal OD_610_ value in the sixth stage, surpassing all other microdroplets (Figure 2, Table 1). Consequently, this top microdroplet was selected as the optimal one. Importantly, the entire MMC-based screening process was completed in 405 h, representing a significant improvement over the conventional plate-based screening method. The system was fully automated, substantially reducing labor workload and consumable usage, while also shortening the overall experimental duration [24].

### 2.3. Direct Isolation of Target Mutants on Dual-Inhibitor Selective Agar Plates

**Figure 3:**
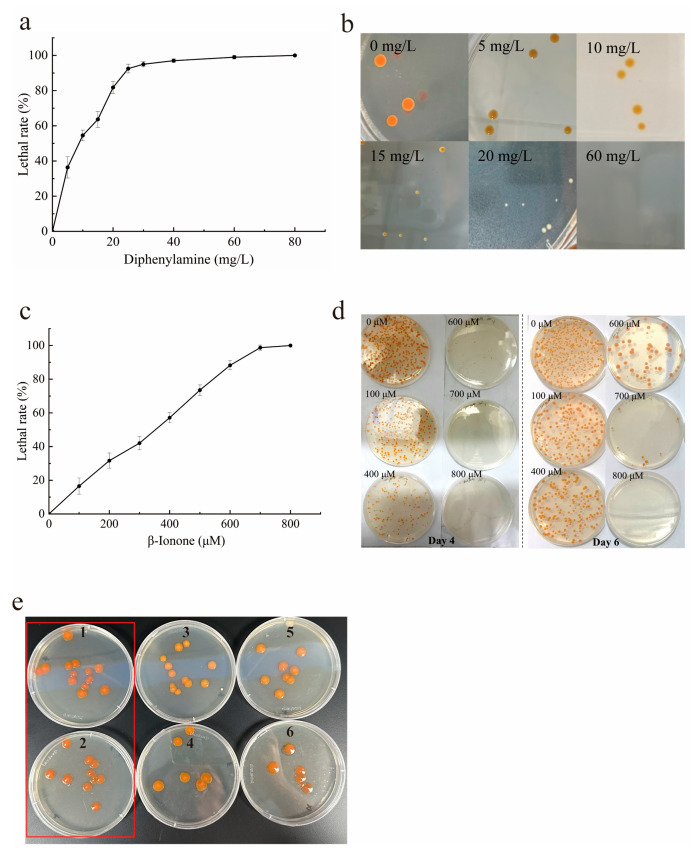
Lethality and growth of *P. marcusii* CGMCC 1.8602 on inhibitor-containing agar plates. (**a**) The lethal rate on diphenylamine (DPA) agar plates. (**b**) Colony growth on agar plates with different DPA concentrations. (**c**) The lethal rate on β-ionone agar plates. (**d**) Colony growth on β-ionone agar plates on days 4 and 6. (**e**) Isolation of single-clone mutants from microdroplet-10 collected in the MMC system on dual-inhibitor agar plates (1–6). Error bars in panels (**a**,**c**) represent standard deviations from three independent experiments. The red rectangular frame indicates the plates from which the mutant strains were primarily screened.

To obtain the target mutants with enhanced astaxanthin capacity, monoclonal isolation was conducted using a selective agar plate with dual inhibitors, after separately determining the optimal concentrations of diphenylamine and β-ionone in LB agar plates. As illustrated in Figure 3a, within the diphenylamine concentration range of 0–25 mg/L, the growth inhibition rate of the strains increased linearly, reaching approximately 95% at 25 mg/L, with nearly complete inhibition observed at 60 mg/L. As the diphenylamine concentration increased, colony diameters decreased, and pigment deposition at the edges gradually faded, resulting in lighter coloration. However, the colonies remained circular with smooth surfaces. When the concentration exceeded 15 mg/L, cell growth was significantly inhibited, and at concentrations above 20 mg/L, most colonies appeared white (Figure 3b). Beyond 60 mg/L, almost no growth was detected (Table S4). When diphenylamine was incorporated into the growth medium, only mutants exhibiting enhanced metabolic flux through the astaxanthin biosynthetic pathway, resistance to the inhibitor, or overexpression of genes encoding key enzymes in the astaxanthin biosynthetic pathway were able to overcome the inhibitory effects and sustain both cell growth and astaxanthin synthesis [25]. These resistant, high-yielding mutants typically displayed distinct red or orange pigmentation due to astaxanthin accumulation, enabling efficient visual screening and isolation on the selective agar plates. Therefore, diphenylamine was consistently employed to screen for mutants with potentially improved carotenogenic capacity. For instance, *H. lacustris* mutants with enhanced astaxanthin production were screened using 12 mg/L diphenylamine by monitoring colony color changes (from green to red) on solid culture medium, resulting in a mutant designated DPA12-2, which exhibited a 1.7-fold higher astaxanthin yield than the WT, reaching 47.21 ± 3.30 mg/g DW [26]. Additionally, 40 μM diphenylamine has been used to screen *Phaeodactylum tricornutum* mutants with enhanced fucoxanthin production, leading to the isolation of a mutant exhibiting a 69.3% increase in fucoxanthin content compared to the WT [27]. In this study, to achieve efficient isolation of astaxanthin-overproducing mutants, the optimal diphenylamine concentration was set at 20 mg/L, corresponding to an approximate 90% lethal rate, which was the lowest concentration at which colonies completely lost pigmentation.

Similarly, β-ionone exhibited a pronounced inhibitory effect on cell growth. As the concentration increased, colony diameters decreased, pigment deposition at the edges gradually faded, and colony coloration became lighter (Figure 3d). Within the concentration range of 0–700 μM, the lethal rate increased linearly, reaching approximately 80% at 600 μM and 100% at 800 μM (Figure 3c, Table S4). β-Ionone is a volatile apocarotenoid produced by the cleavage of β-carotene at the 9,10 (or 9′,10′) double bond, retaining one of the terminals β-ionone rings present in β-carotene and related carotenoids such as astaxanthin. It serves as a competitive inhibitor across the carotenoid biosynthesis pathway in all carotenoid-producing organisms, including the tree-bark epiphytic fungus *Xanthophyllomyces dendrorhous* [28]. Consequently, pigment synthesis is impeded, cellular antioxidant capacity declines, and cells become increasingly susceptible to oxidative stress, ultimately leading to cell death. Thus, β-ionone exerts a negative selective pressure that facilitates the isolation of high-astaxanthin-yielding strains [29]. A concentration of 0.03 M β-ionone was employed to screen *Xanthophyllomyces dendrorhous* mutants with enhanced astaxanthin production, yielding a mutant strain designated *X. dendrorhous* M34, which achieved a total carotenoid yield of 602.4 µg/g DW, representing a 110.8% increase compared with the WT [30]. To minimize screening duration and enhance selection efficiency, a β-ionone concentration of 600 μM, corresponding to an approximate 90% lethal rate, was selected for subsequent experiments. In practice, microdroplet-10 collected from the final stages of the MMC system was diluted and spread onto dual-inhibitor selective plates containing 20 mg/L diphenylamine and 600 μM β-ionone. As shown in Figure 3e, single colonies on agar plates 1 and 2 appeared orange-red, with coloration significantly deeper than that of the other plates. Consequently, 9 single-colony mutants exhibiting deeper coloration and larger colony sizes were isolated and designated as M1, M8, M9, M12, M15, M18, M19, M21 and M23 for subsequent evaluation via liquid culture in shake flasks.

### 2.4. Evaluation and Identification of Optimal Mutant via Liquid Culture

**Figure 4:**
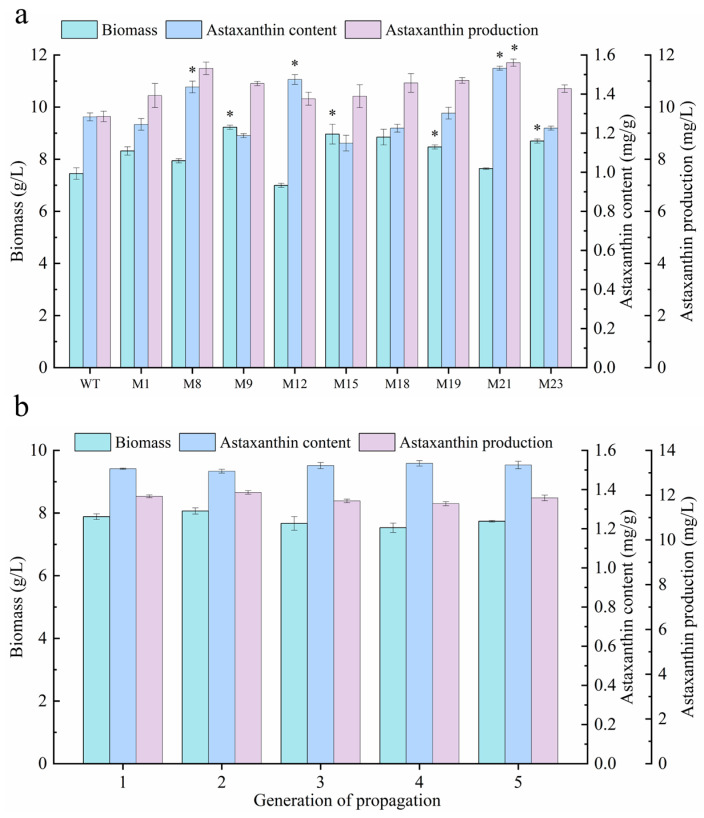
Comparison of biomass concentration, astaxanthin content and production among the wild type and selected mutants of *P. marcusii* CGMCC 1.8602 (**a**); stability of capacity performance in mutant M21 over five generation of propagation (**b**). Asterisks (*) indicate that the mutant strains show statistically significant differences in biomass, astaxanthin content, and astaxanthin yield compared with the WT group (*p* < 0.05).

A total of 9 mutants (M1, M8, M9, M12, M15, M18, M19, M21 and M23) were isolated from the dual-inhibitor selective plates and the WT and were individually inoculated into 100 mL of YEM medium in 250 mL shake flasks. As shown in Figure 4a, the biomass concentrations of M9 and M19 were significantly higher than that of the WT (*p* < 0.05). The astaxanthin content of M8, M12, and M21 was significantly elevated relative to the WT (*p* < 0.05); however, only M21 exhibited a significantly higher astaxanthin production (*p* < 0.05). The biomass concentration of M21 reached 7.64 g/L, which did not differ significantly from that of the WT (7.45 g/L). In contrast, the astaxanthin content and production of M21 reached 1.53 mg/g and 11.71 mg/L, representing increases of 16.86% and 19.81% over the WT, respectively (*p* < 0.05), thereby identifying M21 as the optimal mutant in this study. This was consistent with previous research, where a mutant of *Phaffia rhodozyma* designated Y1 was obtained after being subjected to ARTP mutagenesis followed by diphenylamine screening, resulting in increases in carotenoid concentration and content of 19.02% and 21.20% relative to the WT [23]. This demonstrates that ARTP mutagenesis is an effective strategy for the directed evolution of microbial cell factories [31]. However, the mutagenic efficiency varies among species. For instance, the astaxanthin-producing yeast *S. cerevisiae* showed an 83% increase in astaxanthin levels after ARTP mutagenesis [32], whereas the green microalga *H. lacustris* treated with EMS and UV exhibited 2.38-fold and 2.17-fold increases in astaxanthin content, respectively [33]. Mutant M21 was subsequently propagated for five generations in 250 mL shake flasks. As illustrated in Figure 4b, there were no significant differences in biomass concentration, astaxanthin content or production across the five inoculations, indicating high hereditary stability.

### 2.5. Optimization of Fermentation Conditions for Enhancing Astaxanthin Production

Astaxanthin, as a secondary metabolite, is not essential for cell growth; rather, its biosynthesis is influenced by various stress conditions that are not optimal for cellular proliferation [29]. Consequently, the development of optimal and cost-effective strategies for the large-scale production of astaxanthin is deemed crucial. A total of 29 culture experiments were conducted utilizing a central composite design with three levels across four factors, culture time (d, A), total nitrogen concentration (g/L, B), sodium lactate concentration (g/L, C) and sodium L-aspartate concentration (g/L, D) (Table S2). The experimental design and results (Table S3) were fitted to a second-order polynomial equation. The regression coefficients were calculated, and the fitted equation (in terms of coded values) for predicting astaxanthin production (mg/L) is presented below in Equation (3), irrespective of the significance of the coefficients:

Y = −94.1756 + 24.6079A − 0.2327B − 2.2563C + 2.1380D + 0.2491AB + 0.3994AC − 0.3062AD + 0.0841BC + 0.0440BD + 0.0230CD − 1.6422A^2^ − 0.0806B^2^ − 0.2577C^2^ − 0.0404D^2^ (1)

Herein, Y represents astaxanthin production, and A, B, C, and D represent culture time (d), total nitrogen concentration (g/L), sodium lactate concentration (g/L), and sodium L-aspartate concentration (g/L), respectively.

The coefficient of determination (R^2^) for the fitting Equation (1) is 0.9346, while the adjusted R^2^ (R^2^ _adj_) is 0.8692. The difference between R^2^ and R^2^ _adj_ being less than 0.2, indicating good generalization ability. An adequate precision value of 13.97 was achieved, surpassing the threshold of 4, which signifies that the signal-to-noise ratio is within an acceptable range. Furthermore, the coefficient of variation (CV) was calculated to be 4.85%, which is below the 10% threshold demonstrating low variability and thus high reliability and precision of the measurements.

The ANOVA test is a crucial statistical method used to determine whether there are significant differences among the means of three or more independent groups [34]. The ANOVA results for the regression model concerning astaxanthin production are presented in Table 2. The significance of the variables is indicated by the *F* and *p*-values, where *p*-values less than 0.05 denote significant model terms. A larger *F*-value and a smaller *p*-value indicate a more substantial effect of the corresponding variable [35]. As demonstrated in Table 2, the model was highly significant (*p* < 0.01), and the lack of fit was not significant (*p* > 0.05), suggesting that the response-surface regression model exhibited minimal error and provided an adequate fit for predicting the response. The small difference between the coefficient of determination (R^2^) and the adjusted R^2^ further corroborated the model’s validity for fermentation optimization. The analysis of variance revealed that factors A, B, C, the interaction AB, and the quadratic terms A^2^, B^2^, and C^2^ had extremely significant effects on astaxanthin production (*p* < 0.01), while the interactions AC and BC were also significant (*p* < 0.05). The remaining terms D, D^2^, AD, BD, and CD were not significant (*p* > 0.05).

**Figure 5:**
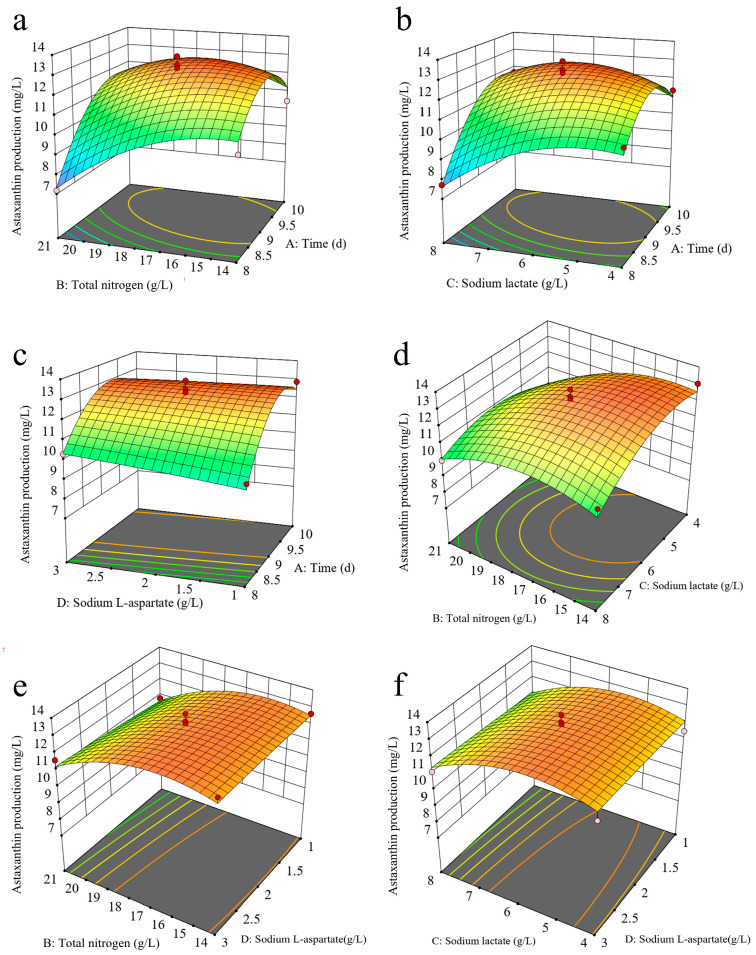
Response surface plots showing the interactive effects of (**a**) culture time and total nitrogen, (**b**) culture time and sodium lactate, (**c**) culture time and sodium L-aspartic, (**d**) total nitrogen and sodium lactate, (**e**) total nitrogen and sodium L-aspartic, and (**f**) sodium lactate and sodium L-aspartic concentrations on astaxanthin production in mutant M21. The Red dots indicate experimental data points, and different colors represent varying levels of astaxanthin production.

The 3D response surface plots were utilized to investigate the interaction of fermentation conditions and to identify the optimal levels that significantly influence astaxanthin production. The response surface plots derived from the model are illustrated in Figure 5. As shown in Figure 5a,b,d, the elliptical contour plots for interactions AB, AC, and BC indicate strong pairwise interactions. Additionally, the steep response surfaces along factors A, B, and C demonstrate their considerable impact on astaxanthin production. In contrast, Figure 5c,e,f illustrate that the contour density along the axes of A, B, and C is significantly higher than that along D, with steep response surfaces for A, B, and C, while D appears relatively flat. These findings confirm that factors A, B, and C have a significant effect on astaxanthin production, whereas factor D does not exhibit a notable impact.

The optimal conditions for maximal astaxanthin production, derived from the differentiation of the quadratic model, are as follows: A = 9.2 d, B = 16.17 g/L, C = 5.48 g/L, and D = 1.95 g/L. The predicted optimal astaxanthin production corresponding to these parameters is 13.08 mg/L. Compared to the conditions prior to optimization, the cultivation time was reduced by 0.8 days, the sodium lactate concentration decreased by 0.52 g/L, and the sodium L-aspartate concentration increased by 0.95 g/L. Sodium L-aspartate can be converted to oxaloacetate through transaminase activity or via a combined deamination reaction with inosine 5′-monophosphate, resulting in the generation of fumarate, which subsequently enters the tricarboxylic acid (TCA) cycle [36]. This process enhances central carbon metabolism, including the Embden–Meyerhof–Parnas (EMP) pathway, the pentose phosphate pathway, and the TCA cycle, thereby increasing the supply of metabolic precursors and ATP generated via mitochondrial respiration. This reduces the overall energy demand for astaxanthin biosynthesis and promotes its accumulation [37]. Lactate can directly enter the metabolic network through the action of lactate dehydrogenase, modulating the flux distribution at the pyruvate and acetyl-CoA nodes. The addition of sodium lactate significantly enhances the flux of acetyl-CoA and its entry into the TCA cycle, thereby promoting astaxanthin synthesis via this cycle. However, excessive sodium lactate exerts an inhibitory effect [13]. For operational convenience, the optimal conditions were adjusted to a culture time of 9 d, a total nitrogen concentration of 16.20 g/L, a sodium lactate concentration of 5.50 g/L and a sodium L-aspartate concentration of 1.95 g/L. To confirm the reliability of the model in predicting maximal astaxanthin production, an additional batch culture of mutant M21 was conducted in triplicates. The average astaxanthin content and production reached 1.72 mg/g and 12.92 mg/L, with deviations of less than 1% from the predicted values, thereby demonstrating the robustness and reliability of the optimized conditions. Under these conditions, M21 exhibited significant increases in both astaxanthin content (12.34%) and production (10.33%) compared to the pre-optimization levels (*p* < 0.01). Furthermore, the optimized conditions enabled M21 to achieve astaxanthin content and production that were 16.44% and 23.02% higher, respectively, than those of the WT (*p* < 0.01) (Figure S1). In addition, the incubation period was reduced by one day, and the concentrations of total nitrogen and sodium lactate used in the optimal medium were markedly decreased, offering clear advantages for cost-effective fermentation.

## 3. Materials and Methods

### 3.1. Bacterial Strain and Seed Culture

*Paracoccus marcusii* CGMCC 1.8602 was purchased from the China General Microbiological Culture Collection Center (CGMCC, Beijing, China) and maintained on LB agar slants at 4 °C [38]. The strain was initially cultivated in 100 mL of YEM medium (Table S1), which contained 20 g/L total nitrogen (yeast extract: beef extract: peptone = 1:2:2, *wt*/*wt*/*wt*) and 30 g/L glucose. Due to the dark brown coloration of YEM agar, which hindered colony visualization, LB agar was utilized for all plate cultures, while YEM served as the medium for liquid culture in shake flask. Prior to inoculation, the pH of the YEM medium was adjusted to 6.5 and sterilized by autoclaving at 121 °C for 15 min (0.1 MPa; GF54DA, Zealway, Xiamen, Fujian, China) [39]. Seed cultures in YEM medium were incubated in 250 mL shake flasks at 25 °C and 160 rpm for 7 days. Log-phase cells on the third day were diluted to a density of 1 × 10^7^ cells/mL (OD_610_ = 1.2) for subsequent experiments.

### 3.2. Condition Optimization of EMS-UV-ARTP Compound Mutagenesis

#### 3.2.1. Ethyl Methanesulfonate (EMS) Treatment

Five milliliters of log-phase culture broth was washed three times with sterile dH_2_O and collected by centrifugation. The resulting pellets were resuspended in 5 mL of phosphate-buffered saline (PBS) containing 0, 50, 100, 200, 300, 400, or 500 mM of EMS, separately. The suspensions were incubated at 25 °C while shaking at 500 rpm for 45 min. To terminate the reaction, 5 mL of 10% (*w/v*) sodium thiosulfate was added to neutralize EMS [40]. The mixtures were then centrifuged, and the pellets were washed three times with sterile dH_2_O, before being resuspended in 10 mL of sterile dH_2_O. The treated cell suspensions were serially diluted, and 0.1 mL aliquots of the appropriate dilutions were spread onto LB agar plates and incubated in an inverted position at 25 °C for 7 days to record colony numbers. All treatments were conducted in triplicate. A lethal rate exceeding 90% was defined as the optimal dosage of EMS treatment and calculated using the following Equation (2):

(2) $$D\;=\;\frac{C_{0}-C_{t}}{C_{0}}\times 100\%$$

where D represents the lethal rate, C_0_ denotes the colony count of the control group, and C_i_ denotes the colony count of a given mutagenic treatment condition.

#### 3.2.2. Ultraviolet (UV) Treatment

Bacterial cell suspensions derived from the optimal EMS treatment were inoculated into 20 mL of YEM medium and incubated at 25 °C with shaking at 160 rpm for 2 days. The resulting culture was diluted to 1 × 10^7^ cells/mL by sterile dH_2_O. Under aseptic conditions, the cell suspensions were transferred into sterile Petri dishes containing magnetic stirring bar and placed on a magnetic stirrer. The cells were continuously exposed to UV irradiation (12 W, 254 nm) from a lamp positioned 20 cm above the liquid surface under light-protected conditions. Exposure times were set at 0, 5, 10, 15, 20, 25, 30, 35, 40, and 60 min. Following irradiation, the cell suspensions were serially diluted, and 0.1 mL aliquots of appropriate dilutions were spread onto LB agar plates for 7 days culture at the same conditions mentioned above [41]. All treatments were conducted in triplicate. A lethal rate exceeding 90% was defined as the optimal duration of UV exposure, as calculated using Equation (2).

#### 3.2.3. Atmospheric Room Temperature Plasma (ARTP) Treatment

Bacterial cell suspensions obtained from the optimal EMS-UV treatment were inoculated into 20 mL of YEM medium and incubated at 25 °C and 160 rpm for 2 days, then the culture was diluted to 1 × 10^7^ cells/mL by sterile dH_2_O. One milliliter of the cell suspension was washed three times with sterile dH_2_O, collected by centrifugation, and resuspended in 1 mL sterile dH_2_O. 10% (*v*/*v*) glycerol solution, sterilized by autoclaving at 121 °C for 15 min, was used as a protective agent. Equal volumes (500 μL each) of the cell suspension and sterile glycerol solution were mixed, and 10 μL of this mixture was evenly spread onto sterile metal carriers. The carriers were subjected to ARTP treatment using an ARTP device (IIS model, Wuxi Tmaxtree Biotechnology Co., Ltd., Wuxi, China) under the following conditions: radio-frequency power at 120 W, 2 mm between the plasma torch nozzle and the sample, helium gas flow at 10 L/min, and exposure times from 0, 20, 60, 80, 100, 120, 140 to 200 s. After treatment, the carriers were transferred into a centrifuge tube containing 1 mL sterile dH_2_O and vortexed for 1 min to elute the cells [19]. The eluates were serially diluted, and 0.1 mL aliquots of appropriate dilutions were spread onto LB agar plates for 7 days culture, during which colony counts were recorded. All experiments were performed in triplicate. A lethal rate exceeding 90% was defined as the optimal time of ARTP treatment, as calculated using Equation (2).

### 3.3. High-Throughput Culture for Screening Fast-Growing Microdroplets in the MMC System

After EMS-UV-ARTP compound mutagenesis under optimal conditions, the eluted cell suspensions from the final ARTP treatment were inoculated into 20 mL YEM medium and incubated at 25 °C and 160 rpm for 2–3 days. The culture was then diluted to an OD_610_ of 0.6–0.8, and 10 mL of the diluted cell suspension was injected into Bottle No. 2 (droplet-generation inlet) of an MMC system (B2L model, Wuxi Tmaxtree Biotechnology Co., Ltd., Wuxi, China), followed by 4 mL of the addition of MMC-specific oil to prevent air exposure. Separately, 10 mL sterile YEM medium was placed in Bottle No. 4 and overlaid with 4 mL of the same oil. Additionally, 10 mL of YEM medium containing 20 mg/L diphenylamine was introduced into Bottle No. 6 and similarly sealed with 4 mL oil [24]. Cell growth in microdroplet was monitored by measuring OD_610_ at 25 °C, and a subculture was performed when the culture entered the late logarithmic phase or reached a maximal OD_610_ of approximately 12. The initial number of microdroplets was set at 100. During multiple runs of cultivation, microdroplets exhibiting poor growth (low OD_610_) were removed, and the remaining microdroplets were continuously sub-cultured. The specific growth rate (μ, h^−1^) was calculated based on OD_610_, and the optimal microdroplets in each run with high growth rates were selected for subsequent subculture or collection. The specific growth rate was determined according to Equation (3):

(3) $$\mu =\frac{\ln\left(\frac{W_{x}}{w_{0}}\right)}{t_{x}-t_{0}}$$

where W_X_ and W_0_ were the OD_610_ at time x (h) and time 0 (h), respectively.

### 3.4. Mutants Isolation on Selective LB Agar Plates

Diphenylamine was dissolved in anhydrous ethanol to prepare solutions, which were added into LB agar medium at final concentrations of 0, 10, 20, 30, 40, 50, 60, 70, 80, and 90 mg/L [17]. Similarly, β-ionone was dissolved in anhydrous ethanol to achieve final concentrations of 0, 100, 200, 300, 400, 500, 600, 700, and 800 μM [42]. All stock solutions were sterilized by filtration through 0.22 μm membranes and subsequently added to sterilized LB agar medium when the temperature was approximately 45 °C. The medium was then poured into Petri dishes and allowed to solidify. The culture broth in the logarithmic growth phase was diluted to a cell density of 1 × 10^7^ cells/mL. Five milliliters of the cell suspension was washed three times with sterile dH_2_O and resuspended in 5 mL of sterile dH_2_O. The resulting cell suspension was serially diluted, and 0.1 mL aliquots of the appropriate dilutions were spread onto selective LB agar plates containing different concentrations of a single inhibitor. Plates were incubated upside down at 25 °C for 7 days, after which colony counts were recorded. The inhibitor concentration resulting in an 80% lethal rate was defined as the optimal dosage, calculated according to Equation (2). Subsequently, selective LB agar plates containing dual inhibitors at the optimal dosages were incubated at 25 °C for 7 days after spreading the diluted cell suspension collected from the MMC system. The visual pigmentation and morphology of single colonies were then evaluated. Colonies exhibiting larger sizes and deeper reddish coloration compared to the WT were isolated as target phenotypic mutants.

### 3.5. Capacity Evaluation and Optimal Mutant Identification

The target phenotypic mutants isolated from selective plates, along with the WT, were inoculated into 100 mL of YEM liquid medium for 7 days culture to further analyze astaxanthin content and production. The mutant with the highest astaxanthin production was designated as the optimal mutant strain. To assess the genetic stability of the selected mutant, both the mutant and the WT were sub-cultured for five consecutive generations on LB agar plates and in YEM medium in shake flasks, respectively.

### 3.6. Optimization of Fermentation Conditions

Based on the results of preliminary experiments, a response surface methodology (RSM)-based experimental design was conducted using YEM medium to further enhance astaxanthin production in the mutant M21, with astaxanthin production (mg/L) serving as the response variable. The factors examined included culture time (8–10 d), total nitrogen (from yeast extract: beef extract: peptone = 1:2:2, *wt*/*wt*/*wt*, 14.0–21.0 g/L), sodium lactate (4–8 g/L), and sodium L-aspartate (1–3 g/L). The factor levels used in the RSM design are summarized (Table S2), where each independent variable assigned three coded levels of −1, 0, and 1. A separate batch culture of mutant M21 was carried out to verify the model’s predictive capability for maximal astaxanthin production under these optimal conditions.

### 3.7. Analytical Methods

#### 3.7.1. Cell Growth and Biomass Production

The bacterial cell concentration of *P. marcusii* was estimated at OD_610_ value of the culture broth [39], as determined by a UV-Vis Spectrophotometer (UV 2300, Techcomp Bio-Equipment Co., Ltd., Shanghai, China). The biomass dry weight (DW) concentration was determined gravimetrically [43].

#### 3.7.2. Astaxanthin

A total of 10 mg of freeze-dried bacterial powder was extracted using 1 mL of acetone, with repeated extractions until the powder appeared white. The pigments in the fixed-volume supernatant were subsequently analyzed using high-performance liquid chromatography (HPLC, 1260; Agilent Technologies Inc., Santa Clara, California USA) equipped with a photodiode array (PDA) detector and a carotenoid column (C30, 150 mm × 4.6 mm, 3 µm; YMC, Kyoto, Japan) [12]. The mobile phases (solvent A, B, and C) consisted of methanol, methyl tert-butyl ether (MTBE), and a 1% phosphoric acid solution, respectively. Gradient elution was performed as follows: 0–15 min, 81–66% solvent A, 15–30% solvent B, and 4% solvent C; 15–23 min, 66–16% solvent A, 30–80% solvent B, and 4% solvent C; 23–27 min, 16% solvent A, 80% solvent B, and 4% solvent C. The flow rate of the mobile phases was set at 0.8 mL/min, and the column temperature was maintained at 25 °C. Detection was performed at a wavelength of 474 nm, and the peaks were characterized based on the retention time and spectrum of the astaxanthin standard (Sigma-Aldrich Chemical Co., St. Louis, MO, USA), with an external standard curve used for quantification.

### 3.8. Statistical Analysis

All experiments were conducted in biological triplicates, and results are presented as the mean ± standard deviation (mean ± SD). Graphs were generated using Origin 2018 (OriginLab, Northampton, Massachusetts, USA), and statistical analyses were performed using SPSS Statistics 26.0 (IBM, Armonk, New York, USA). Orthogonal experimental design and analysis were executed with Minitab 20, while RSM design and analysis were conducted using Design-Expert 13 (Stat-Ease, Minneapolis, Minnesota, USA). Data normality was assessed using the Shapiro–Wilk test, and homogeneity of variances was verified with Levene’s test. Statistical differences between two groups were evaluated using an independent-samples *t*-test. For comparisons involving three or more groups, one-way analysis of variance (ANOVA) was performed, followed by Tukey’s honestly significant difference (HSD) post hoc test. In the figures, asterisk indicates statistically significant differences between groups (*p* < 0.05).

## 4. Conclusions

In this study, an integrated microbial breeding system combining EMS-UV-ARTP compound mutagenesis with the MMC system was established to obtain a novel mutant exhibiting enhanced astaxanthin production. The M21 strain demonstrated a 16.86% increase in astaxanthin content and a 19.81% increase in production compared to the WT. Subsequent RSM-based optimization further elevated the astaxanthin content to 1.72 mg/g and production to 12.92 mg/L, representing improvements of 16.44% and 23.02% over the WT while shortening culture time and reducing total nitrogen and sodium lactate requirements. These findings demonstrate that this integrated strategy holds promise for improving astaxanthin production in *P. marcusii* through sustainable and cost-effective fermentation.

## Figures and Tables

**Table 1:** Specific growth rate (h^−1^) of microdroplets during the sixth cultivation stage in the MMC system based on OD_610_ calculation.

No. Microdroplet	*μ* (h^−1^)	No. Microdroplet	*μ* (h^−1^)	No. Microdroplet	*μ* (h^−1^)
10	0.87	5	0.71	12	0.67
3	0.86	15	0.70	23	0.66
4	0.82	11	0.70	22	0.66
6	0.79	8	0.69	16	0.65
17	0.78	18	0.68	24	0.61
9	0.77	7	0.68	21	0.60
13	0.73	19	0.68	2	0.60
14	0.71	20	0.67	18	0.68

**Table 2:** Regression model of astaxanthin production by ANOVA analysis.

Source	Sum of Squares	df	Mean Square	*F*-Value	*p*-Value	Significance
Model	59.12	14	4.22	14.29	<0.0001	**
A	17.02	1	17.02	57.60	<0.0001	**
B	7.11	1	7.11	24.05	0.0002	**
C	2.67	1	2.67	9.04	0.0094	**
D	0.1984	1	0.1984	0.6711	0.4264	-
AB	3.04	1	3.04	10.28	0.0063	**
AC	2.55	1	2.55	8.63	0.0108	*
AD	0.3749	1	0.3749	1.27	0.2790	-
BC	1.39	1	1.39	4.69	0.0481	*
BD	0.0946	1	0.0946	0.3202	0.5804	-
CD	0.0085	1	0.0085	0.0288	0.8678	-
A^2^	17.49	1	17.49	59.18	<0.0001	**
B^2^	6.33	1	6.33	21.40	0.0004	**
C^2^	6.89	1	6.89	23.31	0.0003	**
D^2^	0.0106	1	0.0106	0.0358	0.8527	-
Residual	4.14	14	0.2956	-	-	-
Lack-of-Fit	3.13	10	0.3134	1.25	0.4487	No significance
Pure error	1.00	4	0.2511	-	-	-
Total sum	63.26	28	-	-	-	-

Note: *, ** represents significant difference (*p* < 0.05) or highly significant difference (*p* < 0.01); A represents culture time; B represents total nitrogen concentration; C represents sodium lactate concentration; D represents sodium aspartate concentration.

## Data Availability

All data are contained within this article and the Supplementary Materials.

## Supplementary Materials

The following supporting information can be downloaded at: https://www.mdpi.com/article/10.3390/md24010019/s1, Figure S1: Comparison of astaxanthin production and content between the WT and mutant M21 after RSM-based optimization; Table S1: Composition of YEM medium in this study; Table S2: Factors and levels design in response surface test; Table S3: Box-Behnken response surface design and results; Table S4: Lethal rate of *P. marcusii* CGMCC 1.8602 on diphenylamine-agar plates and β-ionone-agar plates.
