# Supplementary material for: Enhancing Astaxanthin Production in Paracoccus marcusii Using an Integrated Strategy: Breeding a Novel Mutant and Fermentation Optimization
Source: Mar Drugs. 2026 Jan 1;24(1):19. doi: 10.3390/md24010019 (PMC12842758; doi:10.3390/md24010019)
Supplement: Supplementary file 1 [file marinedrugs-24-00019-s001.zip › marinedrugs-4064291-supplementary.pdf]

Enhancing astaxanthin production in *Paracoccus marcusii* using an integrated strategy: Breeding a novel mutant and fermentation optimization

Yu Li 1,†, Shuyin Huang 1,†, Dong Wei 1,* and Siyu Pan 2

1 Guangdong Province Key Laboratory for Green Processing of Natural Products and Product Safety, Engineering Research Centre of Starch and Vegetable Protein Processing Ministry of Education, School of Food Science and Engineering, South China University of Technology, 381 Wushan Road, Guangzhou 510641, P. R. China

2 Yunnan Asxan Biotech Co., Ltd., 2299 Haiyuan North Road, High-tech industry Development Zone, Kunming 650101, Yunnan, P.R. China

* Corresponding should be addressed to fewd304@scut.edu.cn

† These authors contributed equally to the work and should be regarded as co-first authors.

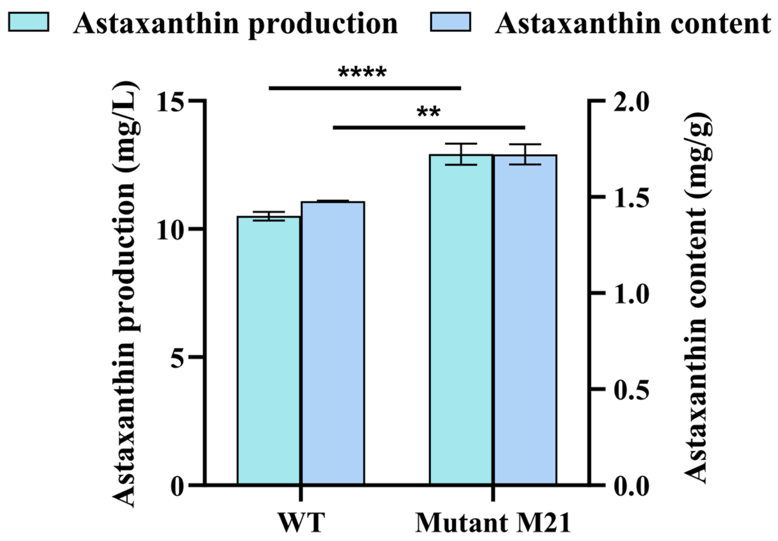

Figure S1 Comparison of astaxanthin production and content between the WT and mutant M21 after RSM-based optimization.

Table S1 Composition of YEM medium in this study.

Components	Concentration (g/L)
Yeast extract	4.0
Beef Extract	8.0
Peptone Bacteriological	8.0
Glucose	30.0
KH_2PO_4	0.19
$MgSO_4 \cdot 7H_2O$	0.7
$CaCl_2 \cdot 2H_2O$	0.05
$Na_2HPO_4 \cdot 12H_2O$	0.48
$FeSO_4 \cdot 7H_2O$	0.3
Biotin	0.5
Sodium aspartate	1.0
Sodium Lactate	6.0

Table S2 Factors and levels design in response surface test.

**Factors and levels**	**A Culture time（d）**	**B Total nitrogen（g/L）**	**C Sodium lactate（g/L）**	**D Sodium aspartate（g/L）**
-1	8	14.0	4.0	1.0
0	9	17.5	6.0	2.0
1	10	21.0	8.0	3.0

Table S3 Box-Behnken response surface design and results.

Run	A	B	C	D	Astaxanthin production （mg/L）		Run	A	B	C	D	Astaxanthin production （mg/L）	
					**Pred.**	**Obs.**						**Pred.**	**Obs.**
1	0	0	0	-1	11.61	11.23	16	0	-1	0	1	11.31	10.94
2	1	0	1	0	10.29	10.31	17	-1	0	0	1	12.19	12.08
3	1	0	-1	0	12.36	12.75	18	0	-1	0	0	13.03	12.56
4	0	0	-1	1	10.00	10.08	19	0	0	0	0	12.99	12.75
5	1	0	0	-1	11.47	12.07	20	1	0	0	0	13.42	12.75
6	0	1	0	0	10.83	10.67	21	0	1	0	0	9.72	9.44
7	-1	1	0	0	7.18	7.29	22	0	1	1	0	9.96	10.57
8	-1	0	1	0	10.45	11.21	23	0	0	1	-1	11.72	12.23
9	0	-1	0	-1	9.76	9.84	24	-1	0	-1	0	12.85	12.44
10	1	0	0	1	10.68	11.03	25	-1	0	0	0	11.70	11.59
11	0	-1	1	0	12.17	12.75	26	1	1	0	0	12.62	12.52
12	0	1	0	-1	10.52	10.16	27	0	0	-1	0	11.16	11.41
13	0	0	0	0	10.92	10.44	28	0	-1	-1	0	12.78	12.47
14	0	0	0	0	12.82	12.75	29	-1	-1	0	0	11.12	11.38
15	0	0	0	0	7.72	7.62	—	—	—	—	—	—	—

Note: Pred. denotes predicted value; Obs. denotes observed value.

Table S4 Lethal rate of *P. marcusii* CGMCC 1.8602 on diphenylamine-agar plates and β-ionone-agar plates.

Diphenylamine (mg/L)	Lethal rate (%)	β-ionone (μM)	Lethal rate (%)
0	36.36 ± 6.00	0	16.53 ± 4.80
5	54.55 ± 3.00	100	31.65 ± 4.50
10	63.64 ± 4.50	200	42.06 ± 4.00
15	81.82 ± 3.50	300	57.14 ± 3.00
20	92.50 ± 2.50	400	73.51 ± 3.10
25	95.00 ± 1.50	500	88.25 ± 2.70
30	97.00 ± 1.00	600	98.71 ± 1.50
40	99.00± 1.00	700	100.00 ± 0
60	100.00 ± 0	800	100.00± 0
80	100.00 ± 0	-	-
